# Why do patients want to have their blood tested? A qualitative study of patient expectations in general practice

**DOI:** 10.1186/1471-2296-7-75

**Published:** 2006-12-13

**Authors:** Marloes A van Bokhoven, Marjolein CH Pleunis-van Empel, Hèlen Koch, Richard PTM Grol, Geert-Jan Dinant, Trudy van der Weijden

**Affiliations:** 1 University of Maastricht, Care and Public Health Research Institute (CAPHRI), Department of General Practice/Centre for Quality of Care Research, PO Box 616, 6200 MD, Maastricht, The Netherlands; 2 Academic Medical Center – University of Amsterdam, Division of Clinical Methods & Public Health, Department of General Practice, PO Box 22660, 1100 DD, Amsterdam, The Netherlands; 3 University of Maastricht, Care and Public Health Research Institute (CAPHRI), Department of General Practice, PO Box 616, 6200 MD, Maastricht, The Netherlands

## Abstract

**Background:**

General practitioners often take their impression of patients' expectations into account in their decision to have blood tests done. It is commonly recommended to involve patients in decision-making during consultations. The study aimed to obtain detailed information on patients' expectations about blood tests.

**Methods:**

Qualitative study among patients in waiting rooms of general practices. Each patient was presented with a short questionnaire about their preferences in terms of diagnostics. Patients who would like blood tests to be done were interviewed.

**Results:**

Fifty-seven (26%) of the 224 respondents wanted blood tests. Twenty-two were interviewed. Patients overestimated the qualities of blood tests. Favourable test results were regarded as proof of good health. Patients regarded blood tests as a useful instrument to screen for serious disorders, and were confirmed in this belief by people in their social environment and by the media. Many patients expected their GP to take an active test ordering approach, though some indicated that they might be convinced if their GP proposed a wait-and-see policy.

**Conclusions:**

GPs' perceptions about patient expectations seem justified: patients appear to have high hopes for testing as a diagnostic tool. They expect diagnostic certainty without mistakes and a proof of good health. The question is whether it would be desirable to remove patients' misconceptions, allowing them to participate in policy decisions on the basis of sound information, or whether it would be better to leave the misconceptions uncontested, in order to retain the 'magic' of additional tests and reassure patients. We expect that clarifying the precise nature of patients' expectations by the GP may be helpful in creating a diagnostic strategy that satisfies both patients and GPs. GPs will have to balance the benefits of reassuring their patients by means of blood tests which may be unnecessary against the benefits of avoiding unnecessary tests. Further research is needed into the effects of different types of patient information and the effects of testing on satisfaction and anxiety.

## Background

Various studies have shown that general practitioners (GPs) regularly order blood tests without good medical arguments [1,2]. Unnecessary supplementary diagnostics have a number of disadvantages. In view of the relatively low a-priori probability of serious disorders in the general practice setting, there is a rather high risk of false-positive test results, which could lead to unnecessary patient anxiety and superfluous further examinations [3,4].

GPs often have non-medical reasons to order blood tests anyway, such as the desire to end the consultation or to reassure a patient, or the assumption that patients expect blood tests or see testing as an indicator of quality of care [5-7]. Although many GPs think that patients expect blood tests, this is not necessarily always true. Some patients mostly expect to be listened to and to get a clear explanation about the nature of their problem, rather than supplementary diagnostics and patients' satisfaction does not appear to be related to being tested [8-10]. Many misunderstandings in the communication between GPs and patients arise from incorrect assumptions about the other's expectations [11]. Good communication requires that GPs are aware of patients' expectations [12]. There have been some quantitative studies into these expectations, which found percentages of patients desiring blood tests ranging from 14 to 22% [13-16]. People tend to greatly appreciate blood tests. A 1995 survey showed that the majority of Dutch people think that an annual medical examination provides hard evidence of their health status, and that nearly all diseases can be cured, provided they are detected at an early stage. People rarely see the disadvantages of screening [17]. There thus appears to be a tension between the frequently limited diagnostic value of blood tests in general practice and the great appreciation for supplementary diagnostics among patients [18]. So far, however, no detailed information is available on patients' motives and their possible misconceptions especially as regards blood tests for diagnostic purposes, the impact of environmental factors on patients' expectations and the role of blood tests in the relation between patients and GPs. Such information would be valuable, as it might provide a basis for patient education. The purpose of the present study was a detailed assessment of patients' views on the value of blood testing when an actual desire for blood tests exists.

## Methods

We conducted a qualitative study in three urban and two rural general practices in the southern part of the Netherlands (n = 17 GPs,), including the following types of practice: one single-doctor practice, three group practices with 3 to 6 GPs and one university-based group practice with 4 part time working GPs. The practices were recruited from a database of addresses of local general practices and the researchers' own network, the aim being to include as many different types of practices, and therefore different patients, as possible. Each practice was visited 1 – 3 times for a full working day by one of the authors (MP). In the waiting room, she invited patients who were at least 18 years old and able to speak Dutch to take part in the study.

Informed consent was obtained to collect the data. If patients refused to take part, only their sex and age category were recorded. All participating patients were presented with a short questionnaire, which asked for demographics and preferences regarding the use of diagnostics by the GP. The following phrases were used: 'Today you have an appointment with your GP. Would you like your GP not only to ask questions but also to do examinations? (yes; no; maybe; do not know). If yes or maybe, please answer question 6' and 'Which examinations would you like to be done? (physical examination (e.g. listening to heart or lungs, examination of your abdomen, blood pressure measurement); blood testing; urine testing; x-ray; scan; echo; otherwise, namely...; I do not know)'.

Patients who answered that they would like to have blood tests done were invited for a semi-structured interview. These interviews were, whenever possible, held before the consultations with the GPs, in order to minimise the influence of the GPs' actions on the patients' views. The interview systematically addressed the following subjects: complaints and ideas about the causes of these complaints, knowledge about and appreciation of blood tests, perceived influences from the patients' direct social environment, GPs and the media on the patients' desire to have blood tests done, reasons for the GP consultations, experiences with blood tests and patient anxiety. Questionnaires and interviews were anonymised to ensure patient privacy. The interviewer recorded the semi-structured interviews on tape and took notes on a structured form during the interview. The interviews were later typed out verbatim.

Two researchers (MB and MP) independently coded patients' answers in all interviews, using a cyclical approach, and then categorised the answers into a number of themes. Data saturation appeared to have been reached after about ten interviews, although the coding of one of the last interviews, with a patient who worked as a nurse, yielded a number of new themes. The codes and themes to be assigned were discussed by the coding researchers until consensus was achieved. Three researchers (MB, MP and TW) further discussed the themes, and categorised them into main topics, which emerged from the data.

## Results

### Population

**Figure 1:**
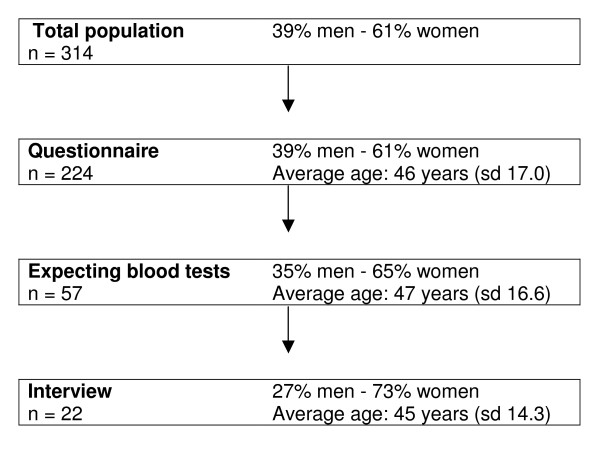
**Flow diagram of patient inclusion**. Sd: standard deviation

Three hundred and fourteen patients were invited to participate in the study, 224 of whom (71%) filled in the questionnaire (figure 1). Of the respondents, 57 (26%) stated they would or might like to have blood tests done. Characteristics of the participants are summarised in table 1. Twenty-eight of the respondents were interviewed, in most cases before the consultation with their GP. The other 29 were not interviewed, for a variety of reasons, mostly because there was not enough time or because several patients were eligible for an interview at the same time. A few patients refused to cooperate in the interview. In the end, 22 interviews were analysed, as six patients were found to have misinterpreted the questionnaire and turned out not to want blood tests at that very moment but at a no specified moment in the future. There were no differences in age or sex between these six patients and those who did want blood tests done.

**Table 1:** Demographic characteristics of participants

n = 224	No blood tests wanted n = 167 (74%)		Blood tests wanted n = 57 (26%)	
Age (mean (SD))	45 (16.8)		47 (16.6)	

Sex	n	%	n	%
Male	66	41	20	35
Female	94	59	37	65

Country of birth	n	%	n	%
Netherlands	139	86	46	81
Other western countries	4	2	6	11
Other countries	13	8	3	5
Unknown	5	3	2	4

Highest level of education	n	%	n	%
Low	27	17	7	12
Middle	102	63	37	65
High	31	19	12	21
Other/unknown	1	1	1	2

Practice setting	n	%	n	%
Urban	137	85	51	90
Rural	24	15	6	11

The patients who were interviewed gave different reasons for consulting their GP, ranging from cardiac complaints to psychological strain, or to be told test results. Four patients had complaints unrelated to their desire to have blood tests done (the latter being done by way of screening).

Most patients thought their complaints were caused by a somatic disorder. Three patients thought their complaints had psychological causes, but nevertheless wanted to have blood tests done to exclude somatic causes.

### Interviews

Three main topic emerged from the data: motives from the patient to be tested, interpretation of results and alternatives to blood test ordering. The results are structured according to these three topics. The three main topics are presented in the headings and the themes within these topics are presented in italics in the text and summarised in table 2. Some illustrative quotations are presented as well.

**Table 2:** Summary of themes in relation to preference for blood tests

Motives	Interpretation of results	Alternatives
+ *indications*: periodical, recurrent or persisting complaints, self-management failed, different pattern of complaints, always, nervous patients, diseases in family, unexplained complaints + *goals*: proving certainty about good health, establishing diagnosis, excluding somatic disorders + *previous experiences*: similar complaints, previous explorative blood tests + *GP should use active policy* + *patient requests blood tests*, capable to indicate need for testing - *patient not taking initiative* + *patient sent by someone else* + *identifying with seriously ill people in environment* + *influence of the media*: reports about young people with serious diseases, information about risk factors for cardiovascular diseases and cancer	+ *tests yield much information*: excluding, early detection, concrete data convince people + *blood tests reliable* + *hardly any false positives and false negatives* + *if abnormal test results do not match patient's presentation*: further examinations required, sign of illness without complaints + *certainty about health status* + *sense of being healthy* + *reassurance*	+ *no wait-and-see*: if risk factors are present, patient worried - *easily convinced by GP: *confidence in GP, satisfied even when expectations are not met - *GP should devote more time and attention* - *preferring explanation to tests* + *hard or impossible to convince: *knowing more about own complaint than doctor, testing necessary for diagnosis, dissatisfied when expectations are not met

+ positive relation with preference for blood tests

- negative relation with preference for blood tests

#### Motives for wanting blood tests to be ordered

The respondents mentioned several *indications *for having blood tests done. They considered it useful to have such tests done periodically, or for recurrent or persistent complaints, when self management failed.

(07103, woman, 19 years)

'Well, yes, in my case it is necessary [to test] because the complaints come back every summer, every spring. So most probably there is something more behind it.'

Another possibility was when the pattern of complaints differed from usual. Some said that tests should always be done if a patient has any complaints, and some felt that there is a greater need for blood tests if patients are nervous or if there are diseases in the family. Many respondents thought that if a GP has no idea about the cause of a particular complaint and is therefore unable to establish a diagnosis (unexplained complaints), blood tests should be done to exclude certain disorders and to reassure the patient.

(18505, woman, 51 years)

' [When you test] you can say concretely: "you do not suffer from that [disease]" and on balance that leads to lower costs for health care and it is a reassurance for the patient. Especially when patients think: "I have a certain disease" you can come up with concrete facts that the blood is all right so nothing is wrong.'

The *goal *of blood tests in their view was to ascertain a person is healthy, to establish the right diagnosis and to exclude somatic disorders, especially cancer and cardiovascular diseases, before discussing psychological causes.

(19003, woman, 21 years)

'I would like to have a test done for all diseases you can possibly get. So that at least you know that you're in good health, that you needn't worry. If your blood is OK, that means you're healthy.'

Respondents who had had *blood tests done before *were more likely to want new blood tests should the same complaints return. If exploratory blood tests had been done before, they wanted renewed tests to monitor aspects like blood cholesterol.

(07103, woman, 19 years)

'I had the same thing a couple of years ago ... they did blood tests then and found that the infection I had also affected the blood. I'm having the same complaints now, so I expect they'll do blood tests this time too.'

The respondents had little to say about their own contributions to the decision whether or not to order blood tests. Some respondents said that they would wait a while before consulting their GP, but would expect the doctor to take an *active approach *during the consultation. Respondents in this group often specifically *asked for blood tests *to be done, whereas there was also a group of respondents who would *not take the initiative *to tell their GP about their preference to have such tests done. One patient, a nurse, said she was capable of indicating the need of lab testing herself because of her professional knowledge.

(22405, woman, 44 years, a nurse)

'No, because I am very convincing. I think that has to do with my background [as a nurse]. And when I think it is not necessary, I don't come. I hammered at it sometime. You visit once and once again and then you think: "this is not right" and then I think: "now I'm going to ask for what I want". And usually they do it then. And I have been correct several times.'

Many of the respondents had discussed their complaints beforehand with someone else, frequently their life partner. Some respondents had been *sent *to their GP *by *this *other person*, as he or she was worried about the complaint or thought they knew the cause. Other respondents had not been influenced by other people's opinions and came of their own accord.

(13103, woman, 36 years)

He [her husband, MB] thought I had had the complaints long enough and he said: "It has been long enough, go and visit the GP".'

Some respondents reported, often spontaneously, that they knew *people who were seriously ill*, and that this had induced them to consult their GP and ask for blood tests.

(06503, woman, 33 years)

*'The main diseases among the people I know are definitely cancer and lung disorders. I also happen to have to acquaintances in their twenties who have breast cancer'*.

The *influence of the media *was evident from the answers by respondents who said they had often read, or seen or heard programmes, about young people with serious diseases such as cancer. These sources also provided them with information about risk factors for cardiovascular diseases and cancer.

#### Interpretation of blood test results

The respondents thought that test results yield *a great deal of information*, and that they can be used to exclude most diseases or detect them at an early stage. The results were seen as convincing because they are tangible. In addition, they supplement the GP's examinations, since a doctor cannot look inside a patient's body.

(08101, man, 61 years)

'The doctor and I are both just ordinary people, and the doctor can't look inside me to see what the matter with me is. That means that blood tests offer additional value in such a situation.'

The respondents thought that *blood tests are reliable*, and that errors are rare, since the tests are done by experts. The occasional incidents were thought to be caused by human errors or equipment errors.

(01401, woman, 41 years)

' [on unexpected results:] 'I hope not, but it's possible. Anyone can make a mistake; people are not perfect. You can never exclude errors completely. There might be one in every so many thousands of tests, I guess?'

*False positive and false negative results *were considered *rare or absent*. Possible causes of unexpected results mentioned by the respondents were the use of medication, having a disease that was not reported beforehand, age, and being tired when blood samples are taken.

(13103, woman, 36 years)

'Yes, when external factors play a role. Like you took medication recently which you did not mention or when you suffer from a disease that you kept silent.'

In addition, false-negative results were thought to result from patients being in an early stage of a disease, in which it is not yet reflected in the blood composition. The respondents did not mention limitations of the tests themselves. Most respondents felt that if a *test result is abnormal *but the patient's presentation does not match this, further examinations are required to find the cause.

(03101, man, 65 years)

'I think that I would go home to discuss and that I would request repeated test ordering some time.'

Finally, they thought that people could be ill without having complaints. This was regarded as more likely than false-positive results.

The effects of blood tests were generally seen by the respondents as highly favourable. They felt that blood tests gave them *certainty *about their health status and gave them a *sense of being healthy*. They often presumed that the tests would show no abnormalities, and regarded normal test results as a guarantee of good health. Normal test results also *reassured *the patients.

(06303, man, 62 years)

I think that if you would blood tests once in a while, you would keep yourself informed about all sorts of things. Because you here such strange things.'

(02501, man, 60 years)

Interviewer: 'why would you like being tested?'

Patient: 'Because I do feel healthy now.'

Interviewer: 'What doe blood test ordering have to do with that?'

Patient: 'Everything: how you're feeling, if you're feeling bad or well. In my case, this blood test ordering is usually all right for me.'

#### Alternatives to blood test ordering

A wait-and-see policy as an alternative to test ordering was often not favoured by the patients. They thought that doctors should *not wait and *see if patients are worried or if there are risk factors for pathology present, such as advanced age or long duration, or high intensity of the complaints.

(10501, woman, 38 years) 'No wait-and-see policy: patient worried'

'I work in health care myself, so I often have a pretty good idea ... And I know it's kind of a problem that goes with the profession, because you see so many diseases in your work. But at the same time I tend to think I have to get it out of my head, and I can only do that by doing something about it. And I want to be able to exclude certain things. I feel like I know my own body and it's giving me a signal that something is wrong.'

When discussing the possibility of alternatives to test ordering, one group of respondents said they would be *easily convinced *if the doctor thought blood testing was not indicated. People in this group had great *confidence *in their GP and were later *satisfied *with the policy adopted by the doctor.

(15302, woman) 'Easily convinced by GP'

*'Sure she can convince me that [a wait-and-see policy] is best. I would not be disappointed in the doctor if she told me that'*.

Some thought it more important that their GP devoted enough *time and attention *to them and *explained things, rather than order blood tests*.

(06503, woman, 19 years) 'GP should devote more time and attention'

'I only expect the doctor to devote enough attention to his patients. Perhaps he should take a bit more time for each patient, as it were, rather than just telling you when you visit him: "Take this or that and come see me again if it doesn't clear up within two weeks". Sometimes the whole consultation lasts only five minutes, which I think is not good.'

The other group said that they would *not be easily convinced, or not at all*, and thought they knew more about their complaint than their GP. These patients thought that a doctor cannot establish an adequate diagnosis without having blood tests done. Correspondingly, they said they would not be satisfied if they didn't get what they expected; they described their dissatisfaction using expressions like 'feeling let down', 'no confidence' and 'not listening to me'.

(14402, woman, 45 years) 'Hard or impossible to convince'

*'She would have to be pretty convincing about her reasons for not doing it ... I'm sure blood tests are not always necessary, but I would expect further examinations'*.

## Discussion

Patients who would like to have their blood tested when they go to their GP tend to have high hopes for blood tests as a diagnostic tool: they assume that such tests yield a great deal of information, that they provide proof of a good health status and that they allow serious diseases to be detected at an early stage without mistakes. When these patients consult their GP, they expect him or her to take an active approach, particularly for complaints for which the doctor is unable to establish a diagnosis as yet. Apparently, according to patients tests are important to provide certainty in situations when the GPs are not capable of providing this certainty themselves. Patient expectations are influenced by opinions of people in their social environment, experiences with serious illnesses among relatives and acquaintances and media information about diseases. Patients in our study often do not appreciate a wait-and-see policy, although a clear explanation by GPs may in some cases make it acceptable to delay diagnostic testing. Patients also reported that they do not like it when their wishes are not met. Patients who have experienced a particular complaint before expect blood tests to be repeated when a new episode of the complaint occurs.

A valuable aspect of our study is that we interviewed patients at a time when they had an active desire to have blood tests done, that is, in the GP's waiting room prior to consultation. We thus collected data not about hypothetical situations but about patients' actual feelings in the real situation. The disadvantage of this timing of the interviews is that we had no time to collect patient details on which we could base a further purposive sampling strategy. As a result we might have missed a few diverging opinions. The 71% of patients who were willing to participate are comparable with the non respondents with respect to sex. This is in line with our impression that response mainly depended on the recruitment strategy. Recruitment by the practice assistant when the patient arrived in the practice yielded a response of almost 100%, while recruitment by the interviewer in the waiting room appeared to depend on the reaction of the first patient: if positive most other patients were also willing to participate, if negative other patients refused as well. We intended to interview all patients who answered on the questionnaire that they maybe or surely wanted to have their blood tested. The aim of the study was to get insight in ideas of patients who did want blood testing in the consultation that day. Therefore this study may not yield a full overview of the determinants of patients' preference for blood tests, since we did not interview people who did not want blood tests to be done at the time. Not all patients who were eligible were actually interviewed. The main reason for this was a lack of capacity of the interviewer so we do not expect this caused biased results. 6 patients appeared to have misunderstood the questionnaire. They did not have a current desire for blood testing on the day of interviewing but would like to be tested some day. Therefore they could not elaborate on the attitudes underlying their desire. In addition, assuming that among the patients who have not been interviewed some also misinterpreted the question, the proportion of patients expecting desiring to be tested diminishes to approximately 20 percent. Since the interviews were treated anonymously, the risk of socially desirable answers was probably small, as is also suggested by the critical remarks made by the respondents. However, triangulation is needed to test the validity of the study's results.

Our findings are in line with the percentages of patients preferring blood testing as mentioned in the literature [12-16]. The perception among GPs that a relevant proportion of their patients, namely about a quarter, do expect blood tests to be done is confirmed. This group wants to be reassured and attaches great, almost magical value to these tests [5]. Kravitz et al also found that patients mention both diagnostic and 'symbolic', to enrich the physician-patient relationship, purposes of testing [19]. An explanation of the great value of tests is that patients see medical techniques as a 'crystal ball' and as an addition to physicians' physical examination skills. Rhodes et al found that diagnostic tests are important to confirming and normalising patients' symptoms, due to historical and cultural factors and the concreteness of the tests, especially when physicians can not locate the problem or are unsure about a solution. They add that patients in that case feel that their complaints are disconfirmed, which may explain the negative expressions of patients we found, if GPs would not order the tests that the patients expected [21]. Our study has revealed a dilemma. The principles of evidence-based medicine require GPs to use their expertise to strike a balance between patients' clinical status and personal circumstances, scientific evidence and the patients' preferences [22]. While the clinical status would often allow fewer diagnostic blood tests to be ordered, and scientific research shows that unnecessary diagnostics have unfavourable consequences, our study shows that many patients still prefer to have blood tests done [23]. Patients do not possess the necessary medical knowledge to make a well-founded choice. The obvious conclusion would be to develop relevant methods to educate patients in this respect, including an explanation of the limitations of supplementary diagnostics. However, as soon as patients have understood and accepted this message, doctors will no longer have the opportunity to use blood tests as a 'magic instrument'. It may be questioned whether this would be a favourable development for patients. On the other hand, deliberately withholding certain types of information from patients could be seen as a paternalistic approach.

## Conclusion

The dilemma of either informing patients about the limitations of tests versus leaving their high expectations of test qualities intact shows that GPs will have to balance the benefits of reassuring their patients by means of blood tests which may be unnecessary against the benefits of avoiding unnecessary tests. By carefully ascertaining the precise nature of patients' request for help, GPs may be able to avoid this difficult choice for a proportion of patients, namely those who will not insist on having blood tests done. This should result in a scenario involving both satisfied patients and a rational diagnostic policy. Further quantitative research is required into (1) the non-diagnostic effects of supplementary blood tests, to allow the advantages and disadvantages of ordering such tests to be balanced and (2) into giving to or withholding from patients information about the limitations of tests.

## Competing interests

The author(s) declare that they have no competing interests.

## Authors' contributions

MB conceived of the study, coded the interviews, interpreted the data and helped writing the manuscript; MP interviewed the patients, coded the interviews, helped interpreting the data and helped writing the manuscript; HK contributed to the conception of the study and critically revised the manuscript; RG supervised the study and critically revised the manuscript; GD helped designing the study and critically revised the manuscript; TW helped designing the study, coordinated the study and helped drafting the manuscript. All authors read and approved the final manuscript.

## Pre-publication history

The pre-publication history for this paper can be accessed here:


